# Healthcare worker training to improve quality of care for sexual and gender minority people in sub‐Saharan Africa: learning from efforts in Uganda

**DOI:** 10.1002/jia2.25728

**Published:** 2021-06-30

**Authors:** Alex S Keuroghlian, Andrew Mujugira, Kenneth H Mayer

**Affiliations:** ^1^ The Fenway Institute Fenway Health Boston MA USA; ^2^ Department of Psychiatry Massachusetts General Hospital and Harvard Medical School Boston MA USA; ^3^ Infectious Diseases Institute Makerere University Kampala Uganda; ^4^ Department of Medicine Beth Israel Deaconess Medical Center and Harvard Medical School Boston MA USA; ^5^ Department of Global Health and Population Harvard T.C. Chan School of Public Health Boston MA USA

**Keywords:** sexual and gender minorities, HIV, education, training, sub‐Saharan Africa, Uganda, PEPFAR

## Abstract

**Introduction:**

Training in care for sexual and gender minority (SGM) populations is critical for ending the HIV epidemic. SGM people, particularly men who have sex with men (MSM) and transgender women, experience disproportionate HIV infection across the globe. The objective of this commentary was to synthesize facilitators of and barriers to SGM health training efforts for healthcare workers in Uganda, in order to help inform potential priorities, strategies and next steps to advance culturally responsive HIV‐related care for SGM communities across Uganda and sub‐Saharan Africa.

**Discussion:**

SGM health training often includes education on: foundational concepts and language; stigma, discrimination and SGM health disparities; understanding and addressing implicit bias; sensitive and effective communication and building SGM‐inclusive and welcoming healthcare environments. Clinicians’ education includes sexual and gender histories, sex‐positive HIV counselling, sexually transmitted infections, HIV pre‐exposure prophylaxis and gender‐affirming hormone therapy. SGM communities in sub‐Saharan Africa have often experienced discrimination, persecution, incarceration and physical violence, and they encounter unique barriers to engagement in sexual health services and HIV prevention and treatment. SGM health training efforts in Uganda reveal challenges to and opportunities for advancing equity for SGM communities in sexual health and HIV medical care across the region. In Uganda, SGM community advocacy, as well as policies and programmes of the Ministry of Health and US President’s Emergency Plan for AIDS Relief, have increased readiness and need for scaling up training and skills‐sharing in SGM‐focused HIV and sexual healthcare, including Ugandan‐led and international initiatives.

**Conclusions:**

Numerous challenges exist to widespread culturally responsive HIV and sexual healthcare for SGM communities in sub‐Saharan Africa. Lessons learned from healthcare worker training efforts in Uganda may inform future replication, adaptation and dissemination initiatives to meet the needs of more SGM communities in the region. Evaluation of SGM health training programmes to determine the impact on HIV virological suppression and sexual health outcomes will be critical for identifying best practices and strategies that may support advancing HIV epidemic control for SGM communities in Uganda and across sub‐Saharan Africa.

## 1 INTRODUCTION

Sexual and gender minority (SGM) people are often stigmatized and mistreated based on their sexual orientation, gender identity or gender expression. Training healthcare workers on serving SGM people is critical for meeting the health needs of these communities, as well as for ending the HIV epidemic. SGM people, particularly men who have sex with men (MSM) and transgender women, experience a disproportionate burden of HIV infection across the globe [1, 2]. In light of the need to engage and retain SGM patients in care to improve virological suppression and decrease HIV incidence, healthcare workers stand to benefit from training to acquire necessary attitudes, knowledge, skills, practices and cultural responsiveness to implement sensitive and effective service delivery for SGM people [3, 4, 5]. Despite significant historical, cultural, economic and sociopolitical differences between healthcare systems in the United States and sub‐Saharan Africa, there are common educational approaches that can be adapted, tailored and evaluated across different countries, with the hope of improving HIV‐related health outcomes for SGM people. The purpose of this commentary is to synthesize facilitators of and barriers to SGM health training for healthcare workers in Uganda, in order to inform potential priorities, strategies and next steps for advancing culturally responsive SGM healthcare across Uganda and sub‐Saharan Africa.

## 2 DISCUSSION

Many in SGM communities have experienced lifelong stressors: pervasive societal stigma in the form of chronic discrimination, victimization and violence that are often associated with adverse mental and physical health outcomes [6]. In the wake of historical and ongoing trauma experienced by various SGM populations attempting to access health services, proactive outreach to local SGM communities requires cultural humility, a necessary first step for health systems to build trust. Local community input helps drive the process of building SGM‐inclusive care environments within sexual health and HIV medical services [4]. One aspect of training healthcare workers to serve SGM communities involves education on basic concepts of sexual orientation and gender identity, sensitive and respectful communication and fostering an inclusive and welcoming care environment [4, 5]. The second component of this training cultivates SGM‐specific clinical skills, such as tailored sexual and gender histories, sex‐positive HIV counselling, sexually transmitted infection (STI) care, HIV pre‐exposure prophylaxis and gender‐affirming hormone therapy [3, 5].

SGM health training is critical for providers who deliver sexual health and HIV medical services, including in sub‐Saharan Africa. HIV incidence and prevalence remain exceptionally high among SGM populations in sub‐Saharan African countries [7, 8, 9]. SGM communities in the region encounter significant psychosocial and structural barriers to engagement in sexual health services, and HIV prevention and treatment [10, 11, 12]. While important differences in experience certainly exist across countries and regional SGM subpopulations, SGM people in several sub‐Saharan African countries have reported discrimination, persecution, forced sex, incarceration and physical violence related to their SGM identities, including government‐led initiatives to suppress local advancement through anti‐SGM policy measures and law enforcement [1, 13, 14, 15, 16, 17, 18, 19].

The experiences of SGM communities vary across sub‐Saharan African countries, and yet SGM health training efforts in Uganda reflect several complex challenges to, and opportunities for, advancing equity in the region with regard to sexual health and HIV medical care. In the context of historical British colonial influence, persistent efforts by U.S. anti‐SGM groups, and perceived imposition of foreign values, SGM Ugandans experience numerous challenges to the protection of their human rights [20]. Colonial‐era anti‐sodomy laws remain on the books [21] and are still intermittently enforced. In 2014, President Yoweri Museveni signed into law the Uganda Anti‐Homosexuality Act that would replace a sentence of life in prison with the death penalty [22]. While the Constitutional Court of Uganda declared this particular law procedurally invalid later that year [23], same‐sex sexual activity remains criminalized and punishable with imprisonment. Over the past decade, anti‐SGM rhetoric by political leaders has been commonplace [24], and police raids of SGM community gatherings in Kampala have occurred on numerous occasions [25]. During this same period, however, a network of Ugandan SGM rights advocates and activists, including the non‐governmental organization, Sexual Minorities Uganda, has emerged and made incremental progress in promoting human rights protections for SGM people through public awareness campaigns and strategic legal actions [26].

The Ministry of Health of Uganda (MOH) issued a statement in April 2014 reminding all Ugandan healthcare service providers not to discriminate against any patients, to ensure maximum confidentiality in all aspects of service delivery, and to limit disclosure of patient information to authorities only for the purpose of improving care [27]. With regard to HIV and sexual health services for SGM people, the MOH has established policies and protocols that include sexual minority men under the framework of key populations (KPs) in HIV care. MSM, along with other KPs such as sex workers, truckers, fisherfolk, people with substance use disorders and prisoners, are eligible within National HIV Testing Services for HIV testing every three months, compared to every 12 months for the general population [28]. KPs are also included within the MOH’s official Assisted Partner Notification Training [29], and MOH‐issued forms to screen for HIV risk and pre‐exposure prophylaxis (PrEP) eligibility include the collection of patient sexual orientation and gender identity data.

Both MSM and transgender women are identified among the categories of KPs in the US President’s Emergency Plan for AIDS Relief (PEPFAR) Uganda Country Operational Plan (COP) [30]. The COP acknowledges that “MSM are highly stigmatized within a legal and policy environment that inhibits non‐discriminatory service delivery […] Transgender Women […] experience high rates of HIV as well as violence […] [and] are marginalized and have barriers to obtaining medical care. Stigma and discrimination against KPs […] in general have been increasing due to the enactment of repressive laws…” MSM and transgender women are prioritized with regard to prevention interventions for epidemic control, including PrEP implementation. PEPFAR Uganda launched a Key Population Investment Fund that calls for all sites it supports to have clinical and non‐clinical staff sensitized on needs of KPs, “including MSM and transgender people,” as well as training health workers in stigma reduction and tailored care for KPs.

Ugandan‐led training exists for healthcare workers in culturally responsive HIV and sexual healthcare for SGM communities, through the Most At Risk Populations Initiative (MARPI) [31]. MARPI is a Ugandan non‐profit organization affiliated with the MOH, which “aims to bridge existing gaps in access to [sexual and reproductive health rights]/HIV/[sexually transmitted infection] services and other interventions among the most at‐risk, key and vulnerable populations in Uganda.” The organization is Uganda’s largest provider of HIV services for KPs, many of whom are SGM people. MARPI has expertise implementing HIV and sexual health services nationally with tailoring for local communities [32, 33, 34, 35, 36, 37], including culturally sensitive HIV counselling and testing, HIV treatment, PrEP, cervical cytology screening and proctology, behavioural change and risk reduction programmes, HIV self‐testing and gonococcal antimicrobial resistance surveillance.

MARPI frames trainings in care for SGM communities by outlining how achieving control of the HIV epidemic requires culturally tailored treatment and prevention strategies, as well as preventing secondary transmission to the general population. The organization emphasizes building capacity in SGM communities through meaningful involvement at all levels of public health planning and implementation under the motto, “Nothing for us without us.” This includes strengthening existing practices to include KP representation on decision‐making committees through the Uganda Country Coordinating Mechanism for the Global Fund, the Uganda AIDS Commission, the MOH and district‐ and facility‐level HIV operations and programming [38, 39, 40, 41].

Based on our experience, MARPI’s educational approach for mitigating adverse effects of anti‐SGM bias on clinical rapport and decision making generally parallels existing training practices in the US [3, 4, 5]. These include: equipping HIV and sexual healthcare staff with the knowledge, skills and empathy needed to effectively engage and serve SGM people; increasing understanding of societal stigma’s relationship to health disparities; cultivating staff’s insight into their own personal level of comfort and confidence serving SGM people; fostering vigilance to keep personal attitudes towards SGM people separate from professional behaviour; applying key concepts and terminology for sensitive and effective communication; promoting warmth and sincerity in service delivery; raising awareness of verbal and nonverbal communication; not trying to change people’s SGM identities and prioritizing flexibility, choice and autonomy in care.

When Ugandan healthcare workers begin intentionally serving SGM communities, cisnormative and heteronormative personal and family values often fuel anti‐SGM stigma and discrimination. This is mostly due to lack of exposure: many Ugandan healthcare workers have never knowingly provided care for SGM people [42]. We have observed that, after receiving training in SGM health, staff often experience gradual changes in attitudes. There is certainly a learning curve as healthcare workers acquire skills in culturally responsive care for SGM people, and mistakes are made along the way. Healthcare workers sometimes proactively ask SGM community members to be patient as teams learn to practice SGM‐tailored approaches. Drawing on SGM community lived experience to teach healthcare workers about the do’s and don’ts of serving SGM people can be very helpful.

The MARPI model of SGM health service delivery, including provider training in cultural responsiveness and involvement of SGM peer educators, has been scaled up nationwide [43]. The MOH, with support from the Global Fund, Community Health Alliance Uganda, and the Joint United Nations Programme on HIV and AIDS, rolled out the MARPI model to several regional referral hospitals in Uganda. Additionally, the MOH has begun training healthcare providers in border areas and along major transport corridors and towns to offer culturally responsive services for SGM communities in specific HIV hotspots.

In 2018, the research team at Makerere University’s Infectious Diseases Institute started conducting SGM‐focused clinical research and adopted the MARPI training model for this purpose. Prior to implementation of the Institute’s first‐ever clinical trial with MSM and transgender sex workers in Uganda, to study the effect of HIV self‐testing on PrEP adherence and sexual risk behaviour [44], the investigators offered MARPI‐led sensitivity training by inviting SGM peer educators to teach research staff about inclusive service delivery. Anecdotally, SGM community partners have reported that the research clinic is inclusive and welcoming, and that they feel safe while there. The research team completed this clinical trial with participant retention rates of 90% for MSM and 81% for transgender women at 12 months, with no reports of social harms to study participants (manuscript in preparation). Building on this effort, the researchers have implemented two new clinical studies with Ugandan transgender women (peer‐delivered combination HIV prevention [45], same‐day PrEP initiation and sexual health self‐care [46]), completed the first study characterizing HIV/STI risk and evaluating PrEP uptake and persistence among Ugandan transgender men (manuscript in preparation), and applied for regulatory approvals to begin the first PrEP clinical trial for Ugandan transgender men. The effective launch of these research studies was critically dependent upon MARPI‐led training in SGM culturally responsive care.

Nevertheless, significant gaps remain in adequately training the Ugandan healthcare workforce to serve SGM people. Notably, to our knowledge gender‐affirming hormone therapy is largely unavailable in Uganda and not specifically recognized or addressed in the existing policy. Clinicians often do not openly prescribe gender‐affirming hormones for fear of losing their licenses. As in the US and elsewhere in the world, key barriers also include a lack of content on gender‐affirming hormone therapy in medical and nursing school curricula. While remaining vigilant about political threats, recruiting US‐based clinicians who could provide basic training for Ugandan gender‐affirming providers may serve as an important next step. As Internet connectivity at rural Ugandan facilities continues to improve, greater accessibility of online live training and technical assistance programmes and no‐cost web‐based educational resources, such The Fenway Institute’s National LGBTQIA+ Health Education Center [47] may facilitate scale‐up of tailored HIV and sexual healthcare for SGM communities, as well as gender‐affirming hormone therapy.

While Ugandan societal views remain largely unchanged, by operating within the confines of existing laws and under the mandate of the Presidential Fast Track Initiative on Ending AIDS in Uganda by 2030 [48], healthcare workers have the potential to offer SGM‐inclusive services despite many prevailing limitations [42]. Given that the MOH prohibits discrimination against SGM people during service provision and is supportive of research with SGM communities, healthcare workers have the chance to forge a narrow operating window. Partnerships with local SGM communities have helped build trust with researchers, and, as a result, the aforementioned peer‐delivered PrEP and same‐day PrEP initiation research studies with Ugandan transgender women have also included Ugandan SGM people as co‐investigators.

Since 2019, PEPFAR and the US Health Resources and Services Administration (HRSA) have partnered with the MOH in a novel clinical skills sharing project, whereby a multidisciplinary team of US clinical experts from HRSA’s Ryan White HIV/AIDS Program (RWHAP) works with local implementing partners to support epidemic control efforts, through in‐person and virtual healthcare facility staff engagement and technical assistance across the Ugandan districts of Jinja, Kayunga and Nebbi [49]. Approaches have included: in‐depth individual and team‐level meetings with local facility staff and community members; staff and community needs assessments and tailored, culturally responsive education, training and capacity‐building activities in collaboration with local staff and peers. This model has enabled the adaptation of US best practices to develop tailored biomedical and psychosocial strategies, including for engagement of and virological suppression among Ugandan KPs. Importantly, this initiative has enabled greater sharing of skills and practices among healthcare workers across Ugandan districts, including over 200 workers from large care teams at 15 PEPFAR‐supported HIV service facilities. Evaluation of the initiative is currently underway, including data collection on processes of training and capacity building, outcomes related to programmatic and service improvements, impact on HIV‐related clinical outcomes and qualitative interviews to further elucidate facilitators of and barriers to implementation. While results from Uganda are forthcoming, previous evaluations of SGM health‐focused training programmes in sub‐Saharan Africa offer encouragement: healthcare worker trainings in both Kenya and South Africa on sexual health needs of MSM were associated with increases in the necessary knowledge and decreases in negative attitudes [50, 51, 52].

## 3 CONCLUSIONS

Numerous challenges exist to widespread culturally responsive HIV and sexual healthcare for SGM communities in sub‐Saharan Africa. Lessons learned in Uganda over the past decade offer insights into potential priorities, strategies and next steps to expand SGM health training for healthcare workers through the engagement of key stakeholders. Culturally responsive health services will necessitate SGM community‐led advocacy, bold MOH and PEPFAR policies with funding for SGM‐led delivery of tailored services, multi‐level meaningful involvement of SGM people in decision making on HIV care, community‐based participatory education, clinical skills sharing across healthcare workers in the region and US clinical experts, and access to free online SGM health education and training resources. Needs assessment and training programme development ought to expand beyond a focus on serving primarily cisgender MSM and transgender women to include care for more SGM populations, such as cisgender sexual minority women and transgender men. Evaluation of SGM health education and training programmes to determine the impact on HIV‐related and sexual health outcomes in sub‐Saharan Africa, coupled with replication and dissemination of evidence‐informed best practices, may hold the potential to facilitate advancement of virological suppression and HIV epidemic control for SGM communities across the region.

## Competing interests

ASK stands to receive future royalties as editor of a forthcoming McGraw‐Hill Education textbook on transgender and gender diverse healthcare. The authors report no conflicts of interest.

## Authors’ contributions

ASK, AM and KHM conceptualized the article. ASK drafted the initial manuscript. AM and KHM edited the manuscript. ASK incorporated all edits and finalized the manuscript. All authors approved the final version of the manuscript.

## Abbreviations

BPHC, Bureau of Primary Health Care; COP, PEPFAR/Uganda Country Operating Plan; HIV, human immunodeficiency virus; HRSA, Health Resources and Services Administration; KP, key population; LGBTQIA+, lesbian, gay, bisexual, transgender, queer, intersex, asexual and all sexual and gender minorities; MARPI, Most At Risk Populations Initiative; MOH, Ministry of Health of Uganda; MSM, men who have sex with men; PEPFAR, President’s Emergency Plan for AIDS Relief; PrEP, Pre‐Exposure Prophylaxis; RWHAP, Ryan White HIV/AIDS Programme; SGM, sexual and gender minority.
